# Engineering a Synthetic Pathway for Gentisate in *Pseudomonas Chlororaphis* P3

**DOI:** 10.3389/fbioe.2020.622226

**Published:** 2021-01-22

**Authors:** Songwei Wang, Cong Fu, Kaiquan Liu, Jiajia Cui, Hongbo Hu, Wei Wang, Xuehong Zhang

**Affiliations:** ^1^State Key Laboratory of Microbial Metabolism, School of Life Sciences and Biotechnology, Shanghai Jiao Tong University, Shanghai, China; ^2^State Key Laboratory of Biobased Material and Green Papermaking (LBMP), Department of Bioengineering, Qilu University of Technology, Shandong Academy of Sciences, Jinan, China

**Keywords:** *Pseudomonas chlororaphis* P3, gentisate, biosynthesis, plasmid-free, bioactive compounds, cell factory

## Abstract

*Pseudomonas chlororaphis* P3 has been well-engineered as a platform organism for biologicals production due to enhanced shikimate pathway and excellent physiological and genetic characteristics. Gentisate displays high antiradical and antioxidant activities and is an important intermediate that can be used as a precursor for drugs. Herein, a plasmid-free biosynthetic pathway of gentisate was constructed by connecting the endogenous degradation pathway from 3-hydroxybenzoate in *Pseudomonas* for the first time. As a result, the production of gentisate reached 365 mg/L from 3-HBA via blocking gentisate conversion and enhancing the gentisate precursors supply through the overexpression of the rate-limiting step. With a close-up at the future perspectives, a series of bioactive compounds could be achieved by constructing synthetic pathways in conventional *Pseudomonas* to establish a cell factory.

## Introduction

Growing attention to environmental problems and energy crises has inspired the development of bio-based production of valuable bioproducts over the past few decades (Choi et al., 2015; Liao et al., 2016; Noda et al., 2017). Microbial-based synthetic biology and metabolic engineering are eco-friendly approaches for producing valuable biochemicals from sustainable carbon sources. Hydroxybenzoic acids and their derivatives are widely used as additives in foods, drugs, and cosmetics for antisepsis and flavor preservation, or as a monomer to synthesize bioactive compounds (Wang et al., 2018a; Shen et al., 2020). Besides, hydroxybenzoic acids play essential roles in microbial metabolism by serving as intermediates of the degradation of the aromatic compounds and contributing to the synthesis of various valuable secondary metabolites. Therefore, it is essential to explore the metabolism of hydroxybenzoic acids in microbial hosts to synthesize new natural products and improve the methods for overproduction of valuable compounds.

Recently, *Pseudomonas* has received significant attention in synthetic biology due to its robustness and metabolic versatility (Belda et al., 2016; Wang et al., 2020). Genome database and tools for gene editing make it possible that *Pseudomonas* become a cell factory for bio-industrial application (Poblete-Castro et al., 2012; Wang et al., 2020). *Pseudomonas chlororaphis* P3 (*P*. *chlororaphis* P3) is one phenazine-1-carboxamide (PCN) producing biocontrol strain obtained from *P. chlororaphis* HT66 with multiple rounds of mutation and selection with enhanced shikimate pathway based on the isobaric tags for relative and absolute quantification (iTRAQ)-based quantitative proteomic analysis (Jin et al., 2016). Based on the efficient shikimate pathway and simple cultivation conditions, *P*. *chlororaphis* P3 has been genetically engineered for the synthesis of arbutin (Wang et al., 2018c).

**Figure 1:**
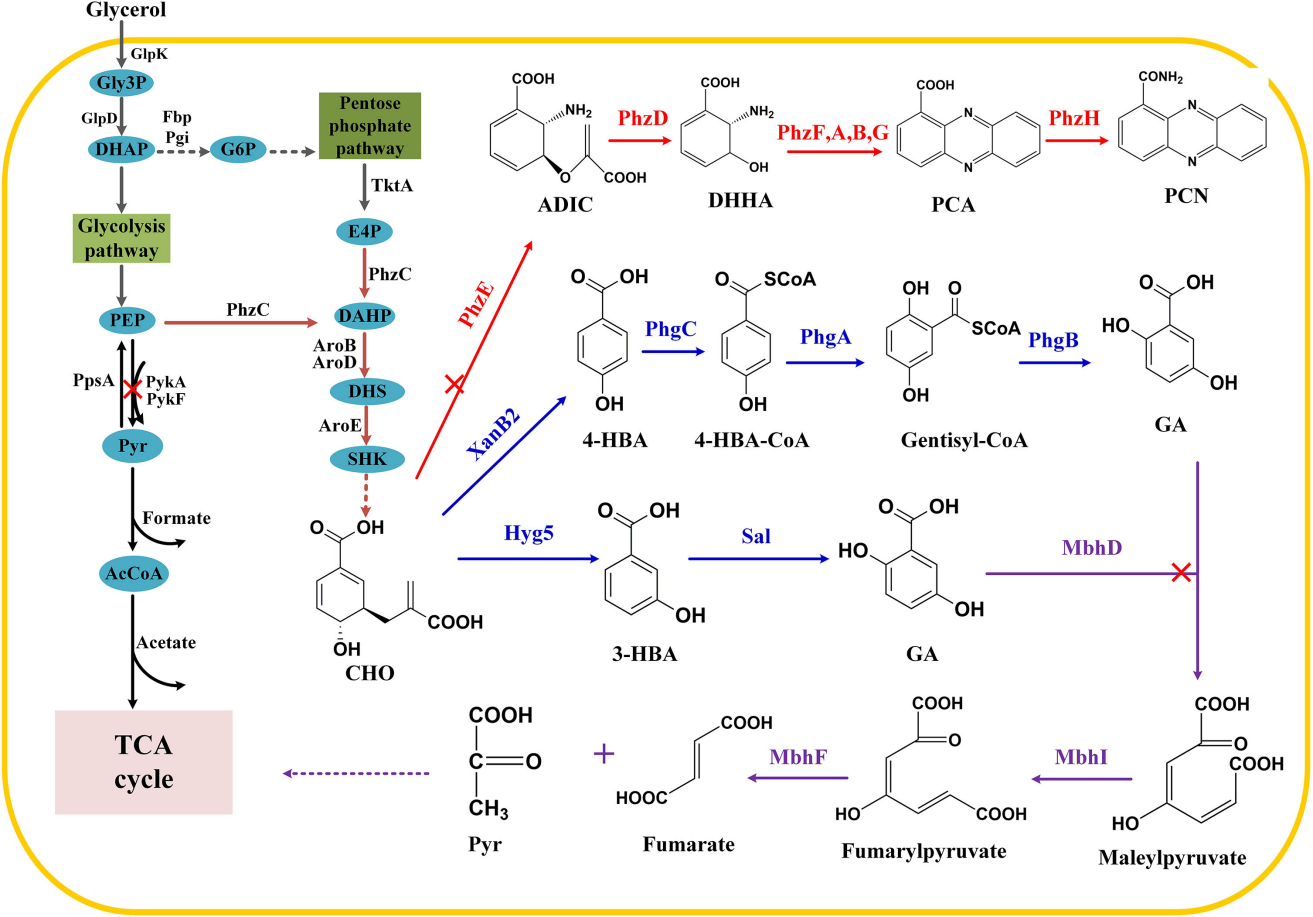
Construction of synthetic pathway for gentisate in *P. chlororaphis* P3. Gly3P, glycerol-3-phosphate; DHAP, dihydroxyacetone phosphate; GAP, glyceraldehyde 3-phosphate; G6P, glucose-6-phosphate; Pyr, pyruvate; PEP, phosphoenolpyruvate; E4P, erythrose 4-phosphate; DAHP, 3-deoxy-D-arabino-heptulosonate 7-phosphate; DHS, 3-dehydroshikimate; SHK, shikimate; CHO, chorismate; 4-HBA, 4-hydroxybenzoate; 3-HBA, 3-hydroxybenzoate; ADIC, 2-amino-2-desoxyisochorismic acid; DHHA, trans-2,3-dihydro-3-hydroxyanthranilic acid; PCA, phenazine-1-carboxylic acid; PCN, phenazine-1-carboxamide. Main enzymes invloved, TktA: pyruvate synthase; PhzC DAHP synthase; Hyg5: 3-hydroxybenzoate synthase; Sal: salicylate hydroxylase; PykA PykF: pyruvate kinase; PpsA: PEP synthase; XanB2: chorismate lyase; PhzE: anthranilate synthase; PhzDFABD: phenazine synthetic protein; MhbD: gentisate 1,2-dioxygenase; MhbI: maleylacetoacetate isomerase.

Shikimate pathway is the leading pathway for the synthesis of numerous aromatic compounds. Besides the aromatic amino acids, folic acid, ubiquinone, and phenazine antibiotics are also synthesized through the shikimate pathway (Averesch and Krömer, 2018; Wang et al., 2018b; Cao et al., 2020). There have been many researches focused on the synthesis of hydroxybenzoic acid and its derivatives, such as the construction of cell factory based on 4-hydroxybenzoic acid (4-HBA) for the synthesis of arbutin, muconic acid (MA), vanillyl alcohol, and other value-added products (Bai et al., 2016; Chen et al., 2017c; Wang et al., 2018c). In addition, salicylic acid (SA) and MA could also be synthesized by introducing isochorismate synthase in *E. coli* (Lin et al., 2014). Gentisate (GA) is an important intermediate with high antiradical and antioxidant activities that can be used as a precursor for drugs. According to an earlier study, plasmid-based 3-HBA expression systems were established that use antibiotics and inducers to ensure the hereditary stability of engineered strains (Kallscheuer and Marienhagen, 2018; Zhou et al., 2019), thus leaving environmental footprints (Keen and Patrick, 2013). In this context, we engineered chromosome-integrated synthetic pathways for 3-HBA and GA in *P*. *chlororaphis* P3. Exogenous 3-HBA synthetic enzyme was introduced, and then GA was biosynthesized by connecting the endogenous degradation pathway from 3-HBA. Also, we tried novel GA synthesis from 4-HBA (Figure 1). Strategies used in this research revealed *Pseudomonas*' versatility as a bioengineering strain, and this green microbial synthetic approach demonstrated its great potential of relieving environmental problems.

## Materials and Methods

### Bacterial Strains, Plasmids, and Culture Conditions

All strains and plasmids constructed or used in this study are listed in the Supporting Information (Supplementary Tables 1, 2). *E. coli* and *P. chlororaphis* were cultured in Lysogeny Broth (LB) medium (Tryptone 10 g/L, Yeast extract 5 g/L, NaCl 10 g/L) during the construction of strains. King's medium B (KB) (Glycerol 18 g/L, Tryptone 20 g/L, MgSO_4_·7H_2_O 1.498 g/L, K_2_HPO_4_ 0.514 g/L) was used for secondary metabolites production in *P. chlororaphis*. Agar was supplemented at a final concentration of 1.5% before sterilization. The medium was supplemented with the following antibiotics: 50 mg/L kanamycin and 100 mg/L ampicillin for screening of positive clones. To induce a double exchange of homologous recombination, sucrose was added to a final concentration of 15% before sterilization. *P. chlororaphis* was cultured at 28°C, while *E. coli* was cultured at 37°C. The shake flasks were filled with 60 mL medium and maintained at 28°C, 220 rpm.

*P. chlororaphis* strains were activated on KB agar medium and cultured overnight at 28°C. Single colonies were isolated and then inoculated to ~50 mL KB medium in a flask. The primary pre-cultures were incubated at 28°C overnight. At the beginning of fermentation, the bacterial suspension was inoculated into a 250 mL shake flask containing 60 mL KB broth to reach an initial OD_600_ of 0.02. Samples of 1 mL were collected every 12 h for the determination of cell growth and metabolic products. Each fermentation test was conducted in triplicate.

### DNA Techniques

All primers were designed by Primer Premier 5.0 (PREMIER Biosoft, San Francisco, USA), and then synthesized by Personalbio (Shanghai, China) (Supplementary Table 3). A sequence of *hyg5* from *Streptomyces hygroscopicus* ATCC 29253 was codon-optimized and synthesized by Genewiz (Suzhou, China). To construct plasmid for expressing *hyg5* in *pykA* locus, the 500 bp upstream and downstream DNA fragments of *pykA*, P_*phz*_ promoter, and open reading frame (ORF) of *hyg5* were amplified by PCR using PrimerSTAR Max DNA Polymerase. Then, the PCR products were purified with HiPure Gel Pure DNA Mini Kit (Magen, Guangzhou, China) after agarose gel electrophoresis. Next, these DNA fragments were assembled into the restriction enzyme-digested pk18*mobsacB* using In-Fusion Cloning Kit (TaKaRa Bio, Beijing, China). After transformation, plasmids were collected by HiPure Plasmid Micro Kit (Magen, Guangzhou, China). Gene deletion or substitution plasmids were constructed using the same method as reported previously (Wang et al., 2018b). The corresponding nucleotide sequences are presented in Supplementary Table 3.

The principle of strain construction is homologous recombination-mediated by suicide plasmids. Here, *pykA* deletion was taken as an example. Similar to early study (Wang et al., 2018b), plasmid pk18-Δ*pykA* containing *pykA* upstream and downstream fragments were constructed using the method mentioned above, and then transferred into S17-1 (λ pir). S17 containing pk18-Δ*pykA* and P3 were inoculated into LB liquid medium and then cultured overnight. To maintain the stability of plasmid, 50 mg/L Kan was added into the medium. A few milliliters of cell suspension were then centrifugated, and the bacteria were mixed with LB liquid medium. After incubation at 28°C for 1–2 h, the mixture was incubated again on a LB solid medium plate at 28 °C for 24–36 h. The mixed bacterial cells were scraped from LB plate, resuspended in 200 μL LB liquid medium, coated on a new LB plate containing 50 mg/L Kan and 100 mg/L Amp, and incubated at 28°C. A single colony was selected, diluted with LB liquid medium to a specific proportion, and then coated on a LB plate containing 15% sucrose. After 36 h of culture, the colonies were selected and cultured on LB plates containing Kan or Amp. The colonies that grow on LB (Amp) plates but not on LB (Kan) plates are positive transformants. To screen the strains, PCR was conducted using pykA-1F and pykA-2R primers. After agarose gel electrophoresis, two or three suspected mutant strains were determined and cultured overnight. Genomic DNA was extracted by HiPure Bacterial DNA Kit (Magen, Guangzhou, China) and used as the template of PCR amplification to verify *pykA* deletion, and no mutation occurred in homologous arms. In this way, a *pykA*-deleted strain was successfully constructed.

### Whole-cell Transformation

BL21 (DE3) strains (i.e., BL21-Sal, BL21-PobA, and BL21-PobAM) were activated on LB plates, cultured overnight at 37°C, and then inoculated to 60 mL LB medium in shake flasks. Isopropyl-β-D–thiogalactopyranoside (IPTG) was used to induce 60 mL cultures at OD_600_ of 0.2–0.6. After overnight incubation at 16°C, the cells were gathered by centrifugal precipitation, washed with 50 mM phosphate buffer (pH = 8.0), and resuspended in 30 mL phosphate buffer at a final OD_600_ of 5.0. Following the addition of 30 μg NADH and 15 mg 3-HBA, the cell suspensions were incubated at 28°C with continuous shaking at 220 rpm. All samples were collected for analysis every 6 h.

### Analytical Methods

The absorbance of cell suspension at 600 nm was determined with an ultraviolet spectrophotometer to measure the number of cells. High-performance liquid chromatography (HPLC) method was established for determining the contents of metabolites. Samples were collected during fermentation at specific time points and then centrifuged at 12,000 rpm for 5 min. Subsequently, the supernatant was filtered using nylon filters with an aperture span of 0.2 μm. The samples of 3-HBA and GA were detected using an Agilent Technologies 1,260 Infinity HPLC system with a C18 reversed-phase column at 30°C and 1 mL/min (constant flow rate). The concentrations of products were determined using an ultraviolet absorbance detector at 235 nm, and the injection volume was 20 μL. The mobile phase consisted of solvent A (methanol) and solvent B (water containing 0.1% formic acid). The separation of metabolites was carried out via gradient elution under the following conditions: 0–2 min, 5% A; 2–10 min, a linear gradient of A from 5 to 15%; 10–20 min, a linear gradient of A from 15 to 25%; 20–25 min, a linear gradient of A from 25 to 30%; and 25–35 min, 5% A.

### Statistical Analysis

All results of three independent experiments were averaged and presented as mean ± standard deviation (SD). Statistical differences among the means of two or more groups (*p* < 0.05) were determined using a one-way analysis of variance (ANOVA) followed by Duncan's multiple range test (SAS Institute Inc., Cary, NC, USA). The number of cells was monitored by measuring OD_600_ values, and the growth curve was fitted with a sigmoidal model. Quantification of the released compounds was performed according to the standard curve calibrated using each authentic compound.

### Sequence Data Analysis

DNA sequences of the genes in *P. chlororaphis* were retrieved from the *Pseudomonas* Genome Database (http://www.pseudomonas.com/). Sequence homology searching was conducted using the NCBI nucleotide BLAST server. The amino acid sequences of 3-hydroxybenzoate 6-hydroxylases and salicylate hydroxylases from other strains were obtained from GenBank. The phylogenetic tree was constructed by MEGA 7.0 using the Neighbor-Joining method.

## Results

### Tolerance of *P. chlororaphis* to 3-HBA and Gentisate

**Figure 2:**
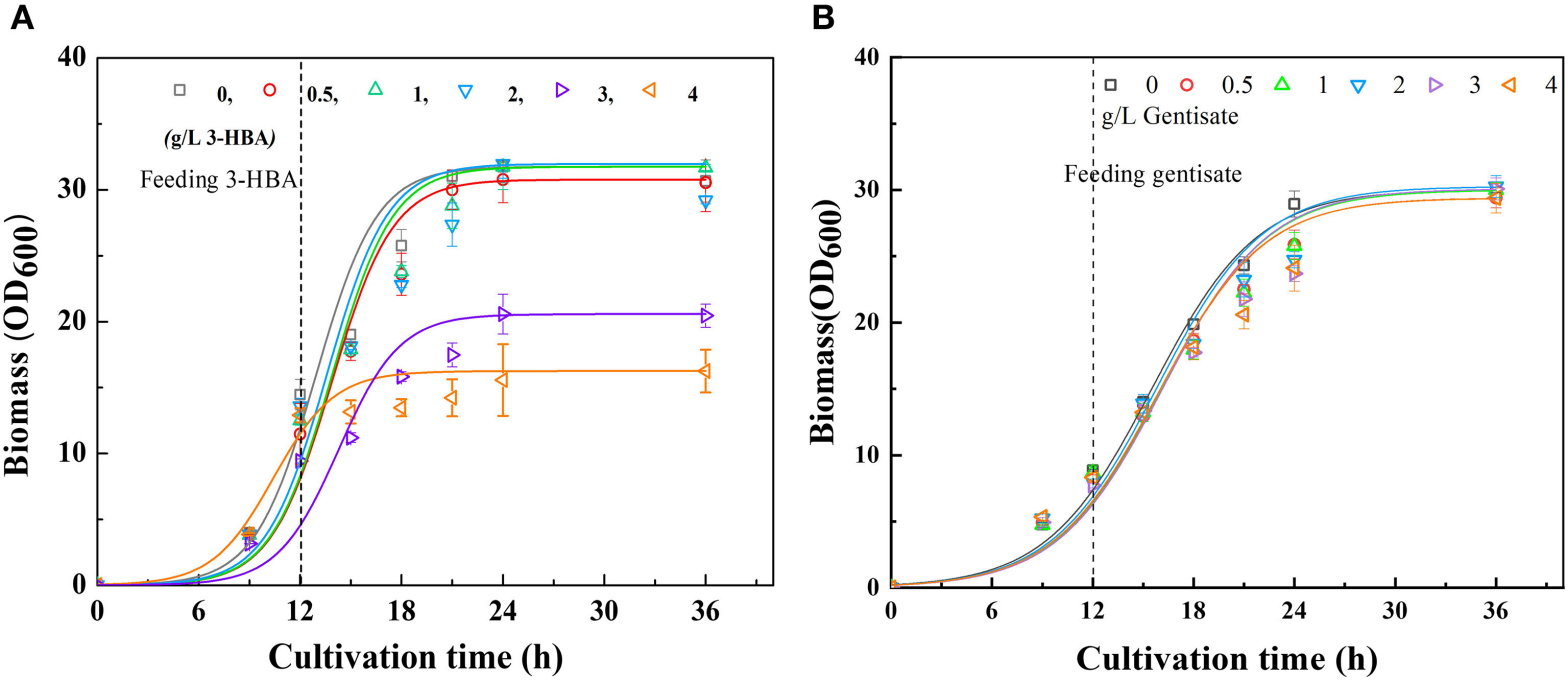
Culture profiles of *P*. *chlororaphis* in KB medium supplemented with 0–4 g L^−1^ 3-HBA or gentisate at 12 h. **(A)** Time courses of a bacterial cell growth when supplemented with 0–4 g L^−1^ 3-HBA, **(B)** Time courses of a bacterial cell growth when supplemented with 0–4 g L^−1^ gentisate. Data are presented as the mean ± standard deviation of three independent experiments (*n* = 3).

It is well-recognized that the accumulation of some metabolites, especially phenolic compounds, is highly toxic to cells (Adeboye et al., 2014). Therefore, to determine whether the excessive accumulation of 3-HBA and GA could affect cell growth, the tolerability of P3 to 3-HBA and GA were evaluated. The tolerance experiment was carried out in shake flasks to simulate the synthesis of secondary metabolites during fermentation. In consistent with the synthetic process, at the early logarithmic growth phase, different concentrations of 3-HBA and GA were supplemented into the medium at a final concentration of 0.5 to 4 g/L. After that, the cells were continuously cultured, and cell growth was monitored until the stationary phase. The results demonstrated that 3-HBA with a concentration of <2 g/L in medium showed no significant effect on cells' growth. When the concentration of 3-HBA reached 3 g/L, the growth of P3 was significantly inhibited, and cell growth was entirely blocked at a concentration of 4 g/L (Figure 2A). Although 3-HBA shows quite a toxicity to *Pseudomonas*, tolerance will not be a crucial factor in current research. When different concentration (0.5 to 4 g/L) of GA were supplemented to the medium, no significant affect was detected on the growth of *P*. *chlororaphis* P3 (Figure 2B). We can conclude that *P*. *chlororaphis* P3 is a good candidate for GA synthesis from 3-HBA.

### Pathway Construction for the Synthesis of 3-HBA

To synthesize 3-HBA from glycerol in *P. chlororaphis*, efforts were first intensified on the upstream of shikimate pathway for accumulating chorismate. Shikimate pathway begins with the aldol condensation of metabolic intermediates (i.e., PEP and E4P) involved in the central carbon metabolism. PEP is a key central metabolite that mainly responsible for the synthesis of pyruvate catalyzed by pyruvate kinase. Therefore, weakening the conversion of PEP to pyruvate may increase the availability of PEP. As reported in *E*. *coli, pykA*, and *pykF* encoding pyruvate kinases, they are responsible for converting PEP to pyruvate in *Pseudomonas* (Meza et al., 2012). Once the two *pyk* genes were deleted, the flux of pyruvate to acetyl-CoA would be impeded, thus interfering with the normal growth of cells. Therefore, *pykA* deletion was carried out based on previous research (Wang et al., 2018c).

Considering that phenazine is the main competitive secondary metabolite in the shikimate pathway, the synthesis of PCN should be blocked to ensure chorismate's maximum availability for other pathways. The formation of 2-amino-4-deoxychorismate (ADIC) is the first step of phenazine synthesis (Li et al., 2011), and thus *phzE* deletion was carried out in this study. 3-HBA is synthesized from chorismate via a reaction catalyzed by chorismatase/3-hydroxybenzoate synthase. It has been reported that *hyg5*, a 3-HBA synthase gene originated from *Streptomyces hygroscopicus* was inserted into plasmids, allowing *E. coli* and *C. glutamicum* to synthesize 3-HBA (13, 14) efficiently. Consequently, *hyg5* was integrated into the genome, *phzAB* locus under the control of native strong promoter P_*phz*_, resulting in a derivative of P3-Hb0. As expected, P3-Hb0 lost the ability to synthesize PCN; however, 3-HBA was not accumulated in fermentation. When using 3-HBA as a sole carbon source to culture *P. chlororaphis* P3, a little colony growth was observed, indicating that 3-HBA can be degraded in *P. chlororaphis*. According to previous reports, there are two major pathways related to the aerobic degradation of 3-HBA: one is the conversion of 3-HBA to 3,4-dihydroxybenzoic acid (protocatechuic acid) catalyzed by 3-hydroxybenzoate 4-hydroxylase, and the product ultimately enters the protocatechuic acid pathway (Michalover et al., 1973); the other is the para-hydroxylation of 3-hydroxybenzoate to produce GA via 3-hydroxybenzoate 6-hydroxylase, and it enters the GA pathway (Groseclose et al., 1973). Since GA pathway is more common in *Pseudomonas*, sequence alignment was conducted using 3-hydroxybenzoate 6-hydroxylase as a template. Thus, Sal, which is annotated as salicylate hydroxylase in the database, has been identified and postulated to catalyze the degradation of 3-HBA, and then *sal* deletion was performed in P3-Hb0, resulting in a derivative of P3-Hb1.

**Figure 3:**
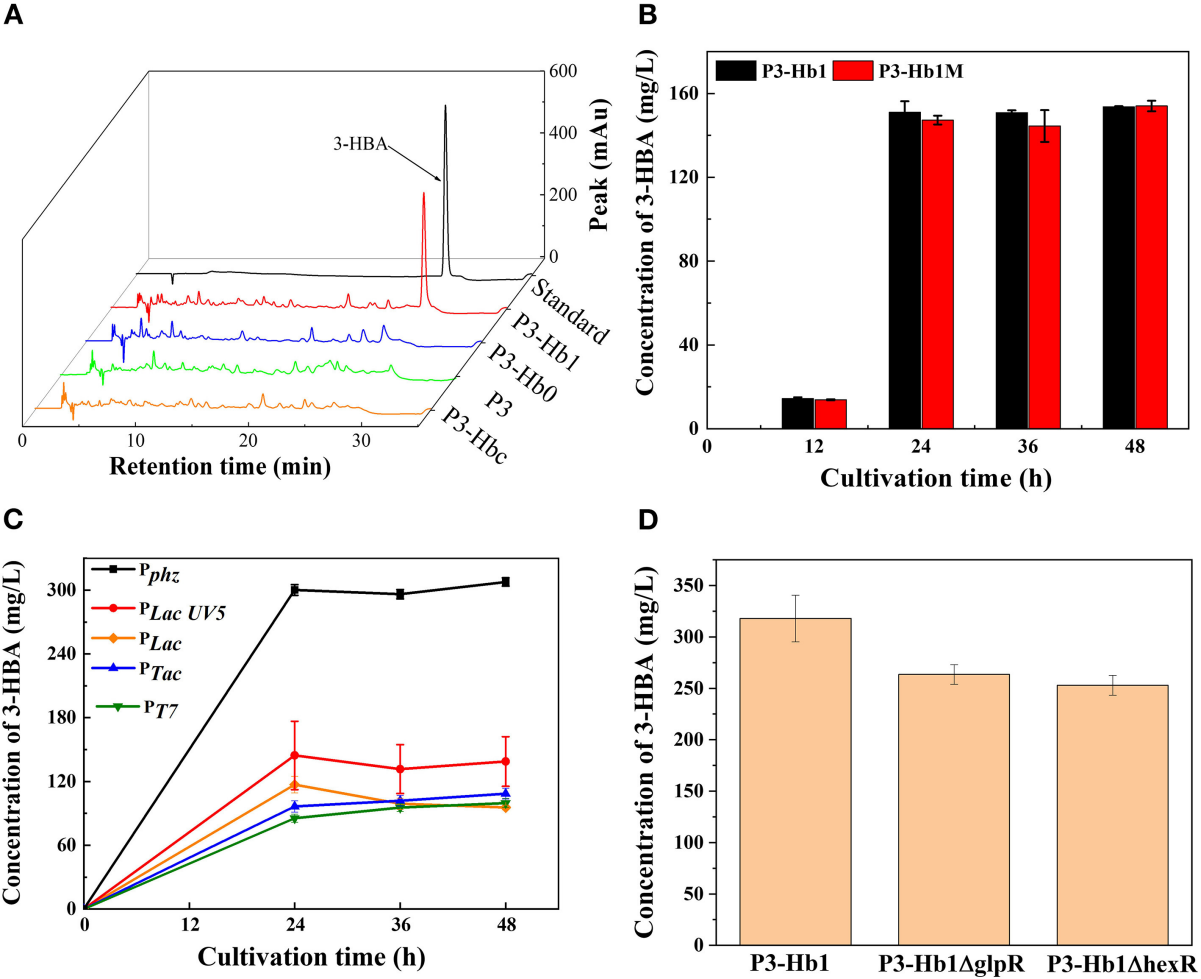
Culture profiles of various 3-HBA-producing transformants. **(A)** HPLC profiles of various transformants; **(B)** The amount of produced 3-HBA in the cultures of P3-Hb1 and P3-Hb1M; **(C)** The amount of produced 3-HBA in the cultures when using different promoter to express *hyg5*; **(D)** The amount of produced 3-HBA in the cultures of P3-Hb1Δ*glpR* and P3-Hb1Δ*hexR*. Data are presented as the mean ± standard deviation of three independent experiments (*n* = 3).

When culturing P3-Hb0 and P3-Hb1 in shake flasks, the products were analyzed by HPLC. As shown in Figure 3, a new peak appeared in P3-Hb1 samples similar to the standard, while no chromatographic peak was found in P3-Hb0 samples over the corresponding time points. To verify the accumulation of 3-HBA, P3-Hb1 sample was further analyzed with ultra-performance liquid chromatography-tandem mass spectrometry (UPLC-MS/MS). The mass spectrometer was operated in the negative ESI mode, and data acquisition was performed in selected-ion-monitoring (SIM) mode. The peak was observed at ~25.6 min, with the *m*/*z* of 137.02, which corresponds to the molecular ion of 3-HBA (Supplementary Figure 1). Collectively, the synthetic pathway of 3-HBA in P3 was successfully established, and the amount of 3-HBA produced from P3-Hb1 was 151 mg/L after 48 h of cultivation.

### Improvement of a Rate-Limiting Step in 3-HBA Production on Multiple Levels

After the successful construction of 3-HBA synthetic pathway, the next step was to enhance the production of 3-HBA. P3-Hb1 produced 151 mg/L of 3-HBA, which was much less than the PCN quantity produced by P3. The supply of precursor chorismate increases the synthesis of 3-HBA, thus it is considered a rate-limiting step that catalyzes chorismate to 3-HBA. To improve the rate-limiting step, we attempted to optimize the expression of genes involved in 3-HBA production at multiple levels.

Firstly, *cuv10*, a candidate 3-hydroxybenzoate synthase gene from *S. hygroscopicus* was used as a substitute for *hyg5*. P3-Hbc was constructed by inserting *cuv10* into its genome under the control of promoter P_*phz*_ for a replacement. As shown in Figure 3A, the chromatographic peak of 3-HBA was not observed in P3-Hbc sample, indicating that *cuv10*-carrying P3-Hb0 cannot efficiently produce 3-HBA.

Secondly, an additional initiator codon sequence was inserted to the upstream region of ORF, *hyg5* possessed double initiator codon in P3-Hb1m consequently. According to a report that the substitution of the start codon to regulate the expression of some genes is one useful strategy in synthetic biology (Chen et al., 2017a). Based on this, we adopted the similar approach to promote the binding of ribosome and mRNA and ultimately increase translation levels. P3-Hb1m was fermented while P3-Hb1 as a control group and then sampled for HPLC analysis. Figure 3B shows the amounts of 3-HBA produced during the cultivation. There was no significant difference in the production levels of 3-HBA between these two strains, and both of them achieved a maximum yield of 151 mg/L after 48 h of cultivation.

Thirdly, *hyg5* was expressed under the control of different promoters. P_phz_ is a native strong promoter located at the upstream of the phenazine gene cluster, which can effectively regulate the transcription levels of the whole gene cluster. Four foreign constitutive promoters (i.e., P_*Lac*_, P_*LacUV*5_, P_Tac_ and P_*T*7_) were cloned from *E*. *coli*, a native promoter (i.e., P_*phz*_) was cloned from *P. chlororaphis*, then linked to *hyg5* and inserted into the genome of P3-Hb1. For construction of P_*T*7_-based expression derivative, T7 RNA polymerase was inserted into the *phzE* locus under the control of native promoter. A total of five derivatives (i.e., P3-Hhb1, P3-Hhb2, P3-Hhb3, P3-Hhb4, and P3-Hhb5) were fermented, and the amount of 3-HBA produced in each derivative is shown in Figure 3C. It was found that P_*phz*_-induced overexpression of *hyg5* could considerably enhance the production of 3-HBA, with a maximum level of 300 mg/L, which nearly doubled compared to P3-Hb1. However, other promoters did not exhibit a positive effect on the improvement of 3-HBA production.

Lastly, global metabolic regulation was concerned. It has been reported that the deletion of *glpR* in *P. putida* could eliminate its growth lag-phase and increase polyhydroxyalkanoates accumulation when cultured on glycerol (Escapa et al., 2013). Besides, the transcriptional factor HexR regulates the central carbohydrate metabolism globally. According to previous findings, pyruvate kinase is regulated by HexR (Leyn et al., 2011). Thus, *glpR* and *hexR* were deleted in P3-Hhb1, individually. Contrary to our expectation, the amount of 3-HBA decreased slightly (Figure 3D). We assumed that GlpR displays positive action on central carbohydrate metabolism in *P*. *chlororaphis*, once deleted, the precusor of PEP is decreased for shikimate pathway, more evidence should be revealed.

### Biosynthesis of Gentisate From 3-HBA

**Figure 4:**
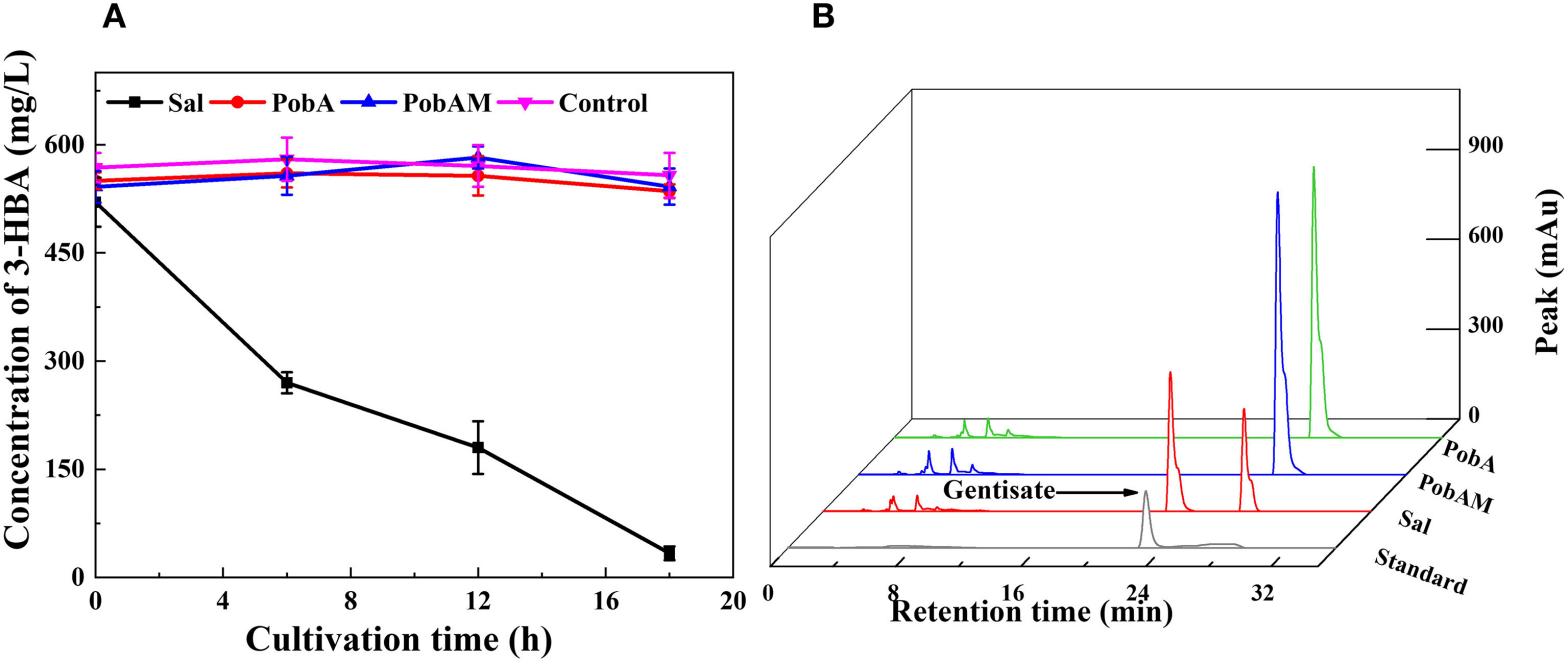
3-HBA consuming profiles by different monooxygenase. **(A)** Time courses of 3-HBA consuming, **(B)** HPLC profiles of various transformants for 3-HBA and gentisata.

As the efficient production of 3-HBA was achieved, we attempted to construct the pathway for GA from 3-HBA. During the 3-HBA synthetic pathway construction, Sal was found to catalyze the degradation of 3-HBA by adding a hydroxyl group to the benzene ring. Therefore, different hydroxybenzoic acid monooxygenases were screened for catalyzing 3-HBA to dihydroxybenzoic acid derivatives. According to a previous report, *p*-hydroxybenzoate hydroxylase encoded by *pobA* from *P*. *aeruginosa* was mutated into Y385F/T294A PobA (hereinafter referred to as PobAM). PobAM displayed a high catalytic activity toward 3,4-dihydroxybenzoic acid and catalyzed the formation of gallic acid (Chen et al., 2017b). Thus, the hydroxybenzoic acid monooxygenase genes (i.e., *sal, pobA* and *pobAM*) were linked to expression vector pET28a(+) and subsequently transferred into BL21(DE3). 3-HBA was added to the cell suspension culture with a final concentration of 500 mg/L, and the concentration changes of 3-HBA in the four groups are presented in Figure 4A. After 12 h of incubation, most of 3-HBA was catalyzed by Sal, while the concentration of 3-HBA did not differ significantly between PobA and PobAM groups. The chromatographic peaks of 3-HBA in the four groups at 12 h are shown in Figure 4B. Results demonstrated that a new substance appeared when catalyzing 3-HBA by Sal. In comparison with the HPLC profile of various hydroxybenzoic acids, it can be speculated that the new substance is GA. Furthermore, UPLC-MS/MS analysis also supported the speculation that Sal can catalyze the conversion of 3-HBA to GA (Supplementary Figure 2).

The degradation pathway of 3-HBA in *Pseudomonas* has also been clarified. As reported earlier, 3-HBA was converted to GA, and then maleylpyruvate was formed by GA 1,2-dioxygenase (MhbD) mediated ring-cleavage reaction. The final products of the above reactions are pyruvate and fumarate, which ultimately enter the TCA cycle (Lin et al., 2010; Wang et al., 2020; Figure 1). The genes encoding these enzymes are all located within a single gene cluster, involving a 3-HBA transporter (Xu et al., 2012; Wang et al., 2020). Thus, a pathway for GA accumulation was constructed by deleting *mhbD1* in P3-Hb0. Notably, the chromatographic peak of GA was observed, implying that GA was accumulated successfully in P3-GA1. Besides, the concentration of GA in fermentation broth reached a maximum of 105 mg/L after cultivation for 24 h and then decreased to 47 mg/L at 36 h.

### Improvement of Gentisate Production

**Figure 5:**
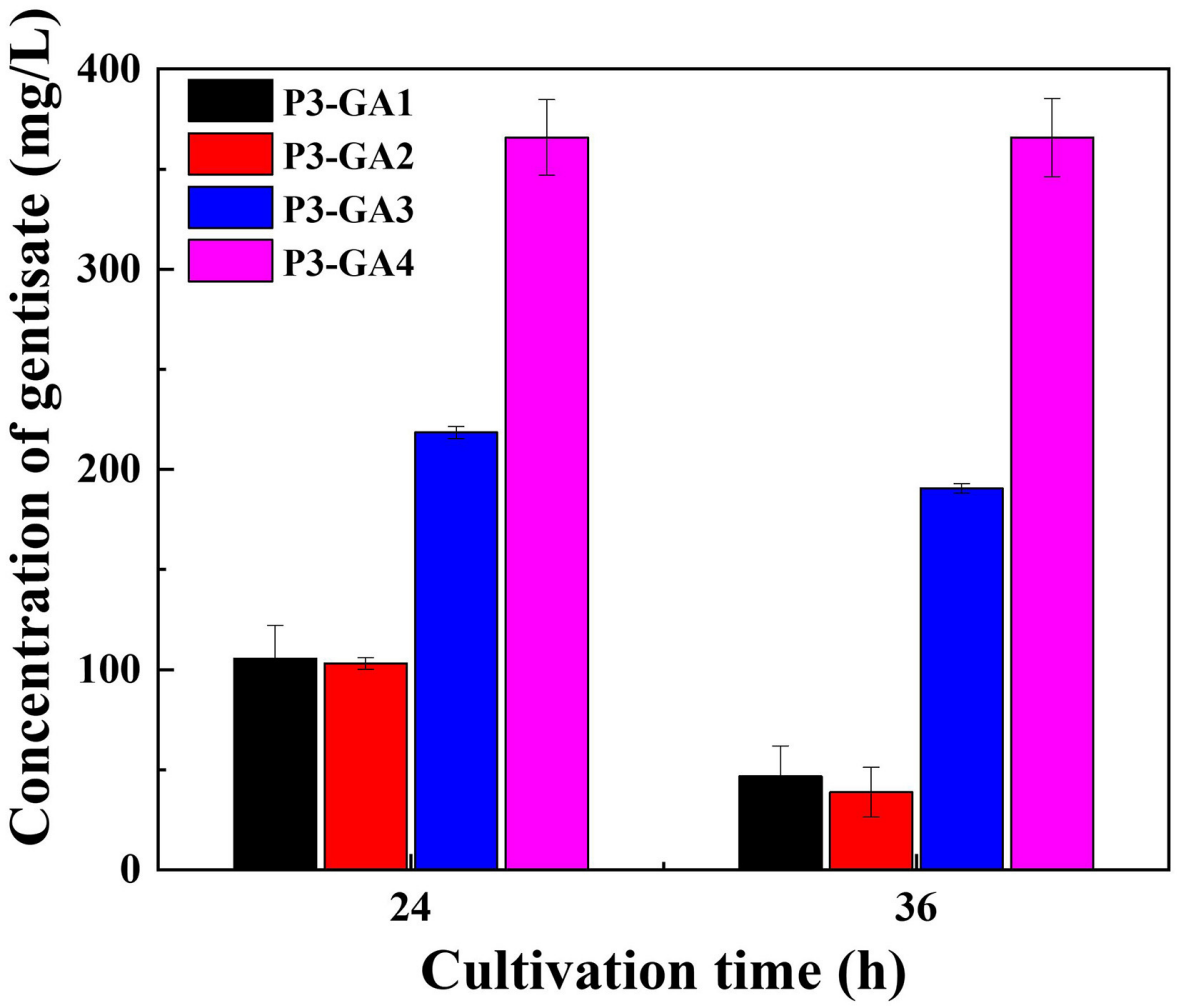
Improvement of rate limiting steps for enhancement of gentisate production.

As mentioned above, GA has accumulated in *P*. *chlororaphis* P3 temporarily, the concentrations of GA were decreased during fermentation, suggesting another degradation pathway for GA in *P*. *chlororaphis*. After database searching, a gene was identified and named as *mhbD2*, due to its position on the antisense strand. It has been recorded to encode a GA 1,2-dioxygenase, according to the *Pseudomonas* Genome Database. When deleted *mhbD2*, the degradation of GA was similar to P3-GA1 (Figure 5).

Increasing the availability of precursors to enhance the production of GA may be an option to offset the degradation. Overexpression of *hyg5* was conducted in P3-Ga0 under the control of P_*phz*_ promoter, resulting in P3-GA3. To our expectation, the amount of GA was doubled to 218 mg/L at 24 h of fermentation. It was noteworthy that the yield of GA at 36 h was 190 mg/L, and the degradation of GA from 24 to 36 h was retarded with its improved production rates. Following *hyg5* overexpression, *hmgA* that encodes homogentisate 1,2-dioxygenase was inactived, since the structure of homogentisate is relatively similar to GA. After deleting *hmgA* in P3-GA3, the amount of GA reached 365 mg/L at 24 h of fermentation, which was 67% higher than P3-GA3. There was no significant difference in the amount of GA between 24 and 36 h, indicating that GA is no longer degraded during fermentation process (Figure 5). Therefore, a stable and effective GA biosynthetic pathway was successfully established in the present study, and 365 mg/L GA was produced in P3-GA4.

### Biosynthesis of Gentisate From 4-HBA

4-HBA has recently emerged as a versatile intermediate for several value-added bioproducts, such as muconic acid, arbutin, gastrodin, xiamenmycin, and vanillyl alcohol using 4-HBA as the starting feedstock (Wang et al., 2018a,c). A novel reaction in the conversion of 4-HBA to GA was reported (Zhao et al., 2018), in which three genes (*phgABC*) catalyze the transformation of 4-HBA to GA via a route involving CoA thioester formation, hydroxylation concomitant with a 1, 2-shift of the acetyl CoA moiety and thioester hydrolysis (Figure 1). Using our earlier screened XanB2 for 4-HBA synthesis, we integrated P_*phz*_-*xanB2* on *pykA* locus, with *phzE, pobA, mhbD1* and *hmgA* deleted. Then, *phgA*-*phgB*-*phgC* were integrated on *phzAB* locus under the control of native strong promoter P_*phz*_, yielding one GA derivative GA-4HBA. When fermented in KB medium, unfortunately, no new peak appeared and no siginificantly 4-HBA reduced.

## Discussion

GA is an important chemical with high industrial values, together with other hydroxybenzoic acids, including salicylic acid, 4-HBA, 3-HBA, and so on. There have been many reports about hydroxybenzoic acid production and their derivatives in various microbial systems (Wang et al., 2018a). Although antibiotic compounds are concerned as 'emerging contaminants' (Keen and Patrick, 2013), the biosynthesis of GA independent of inducers and antibiotics has not been achieved previously. In this work, we engineered a chromosome-integrated synthetic pathway for GA production from 3-HBA in *Pseudomonas*.

3-HBA usually serves as an important platform chemical in microorganisms, in which its synthetic pathway can be found and reconstructed. We focused on the enhanced shikimate pathway in *P. chlororaphis* P3, the leading pathway for aromatic compound synthesis. Apart from introducing exogenous 3-hydroxybenzoate synthase to catalyzes 3-HBA from chorismate, another approach was to prevent the degradation of the products and their potential precursors in *Pseudomonas*. To enhance the synthesis of 3-deoxy-D-arabino-heptulosonate 7-phosphate (DAHP), *pykA* deletion was conducted in P3. Afterwards, *phzE* that catalyzes chorismate to phenazines and *sal* that degrades 3-HBA were knocked out, individually, resulting in a chassis strain for 3-HBA synthesis from glycerol (Figure 1).

To enhance the production of 3-HBA and GA, multiple strategies were employed. Several reports have described that the feedback inhibition in the shikimate pathway may impede the production of target products (Kikuchi et al., 1997; Juminaga et al., 2012). However, on the basis of our previous findings, we speculated that such bottleneck mainly occurs during the conversion of chorismate to 3-HBA in *P*. *chlororaphis* (Wang et al., 2018b). Few 3-hydroxybenzoate synthase genes were reported, and the enzymes with higher activity than Hyg5 are not available for a replacement. According to reports, Cuv10 exhibits maximum activity at 26°C (pH 6.5), the culture conditions of *P. chlororaphis* may not meet the enzymatic properties of Cuv 10. Meanwhile, *cuv10* is a part of a native polyketide synthase gene, and it is probably non-functional as chorismatase in *Pseudomonas* (Jiang et al., 2013).

Moreover, the enhancement of the start codon did not upregulate the activity of *hyg5* The overexpression of *hyg5* under the control of native strong promoter P_*phz*_ significantly upregulated its expression levels, which in turn led to a double increase (300 mg/L) in 3-HBA production (Figure 3C). After that, *glpR* and *hexR* (encoding transcriptional regulator) were deleted to optimize metabolic regulation. The results demonstrated that the metabolic flux to shikimate pathway has been improved in *P*. *chlororaphis* P3, and the deletion of *glpR* and *hexR* might cause an imbalance in primary metabolism and energy flux, resulting in a negative effect on the production of 3-HBA (Figure 3D). Thus, the highest titer of 3-HBA in P3-Hhb1 was 300 mg/L. The reaction of Hyg5 undergoes an intramolecular arene oxide mechanism, starts with surmounting a high energy barrier (Dong and Liu, 2017), thereby resulting in its low level of activity.

**Figure 6:**
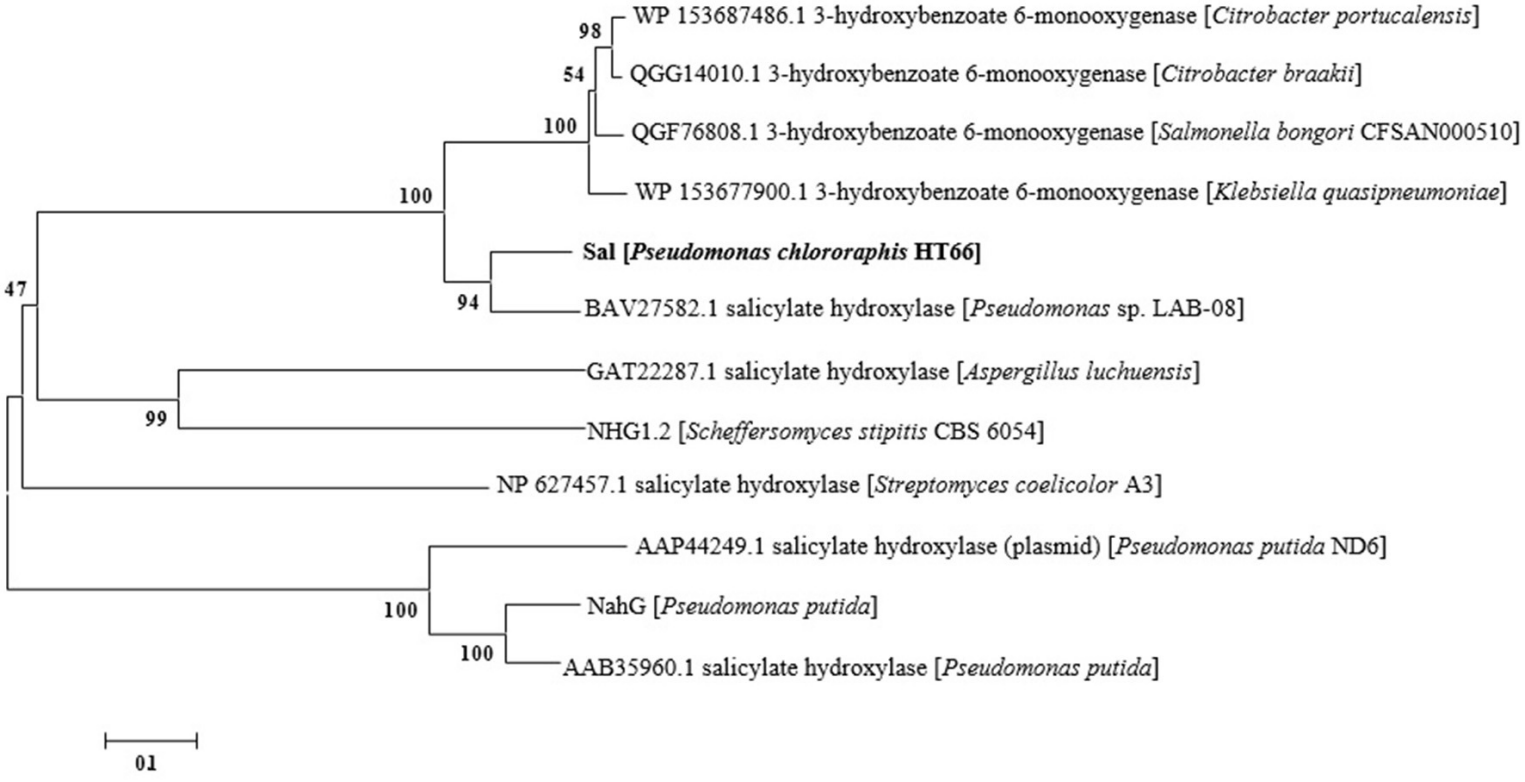
Phylogenetic tree of of 3-hydroxybenzoate 6-hydroxylase, salicylate hydroxylase and Sal. The ruler at the bottom of figure indicates the horizontal distance equal to 10% sequence divergence.

Based on the whole-cell catalysis experiment of 3-HBA, we identified that endogenous Sal catalyzed the conversion of 3-HBA to GA with high efficiency, which displayed the characteristics of 3-hydroxybenzoate 6-hydroxylases. As shown in Figure 6, a phylogenetic tree was constructed with representative 3-hydroxybenzoate 6-hydroxylases and salicylate hydroxylases from other strains (Chen et al., 2018). As shown, Sal reveals as one of these two groups, suggesting that Sal may display unselective substrate adaptability and dual catalytic activity (Fang and Zhou, 2014). Both salicylate hydroxylase and 3-hydroxybenzoate 6-hydroxylases belong to the same family of flavin-dependent monooxygenases (Yang et al., 2011; Huijbers et al., 2014), with high sequence homology between them.

Upon assessing the production of GA, no 3-HBA was found in the samples, indicating that endogenous Sal catalyzed 3-HBA effectively. As mentioned above, the maximum amount of GA was achieved simultaneously (24 h of cultivation) as 3-HBA. However, the net conversion rate of 3-HBA to GA was only 62.2% (the mole ratio) at 24 h without residual 3-HBA, which was much lower than the theoretical conversion rate, indicating that a third of GA was degraded. To eliminate the degradation caused by spontaneous oxidation, GA was added to KB medium and incubated at 28°C for two days. No significant change was detected in the culture, confirming that GA is relatively stable in the culture (Supplementary Figure 3).

GA 1,2-dioxygenase detected in other *Pseudomonas* shared a high homology level with MhbD1. There are few reports of other GA degradation genes. In this study, gene *mhbD2* was identified. Sequence alignment and analysis revealed that *mhbD2* was not associated with the synthesis or degradation of GA, and it probably encoded a member of fumarylacetoacetate hydrolase family protein. Thus, we attempted to enhance the carbon flux from chorismate to GA by overexpressing *hyg5*. Consequently, the maximum amount of GA produced was doubled, and the degradation was partially offset. Interestingly, when *hmgA* was deleted, the production of GA was improved entirely. It has not been reported that homogentisate 1,2-dioxygenase catalyzes GA previously, but it does indeed exist in *Pseudomonas*. Our results suggest that microbial catabolism is not only composed of one single pathway and contains an interrelated metabolic network. The unselective substrate adaptability of Sal and HmgA reflects the versatile metabolism of *Pseudomonas*, indicating that our platform strain P3 has a great potential to synthesize valuable chemicals via metabolic engineering. Unexpected, when *phgABC* were expressed for synthesis GA from 4-HBA based on NIH shift, no significant GA was accumulated. For the lower specific activity of PhgC against 4-HBA (Zhao et al., 2018), the higher concentration of 4-HBA may inhibit the expression of PhgC.

Various pathways could be linked to the shikimate pathway. At present, we have synthesized versatile platform compound GA and 3-HBA from simple carbon sources successfully, and many other GA-based value-added derivatives could be synthesized in the near future. Pathways can be designed to produce gallic acid via hydroxylation of 3-HBA and protocatechuic (Chen et al., 2017b). According to a report, industrially valuable maleate production was attained by extending chorismate and GA pathways (Noda et al., 2017). Besides, the hydrolysis of chorismate to 3-HBA involves the synthesis of macrocyclic polyketides, which has attracted great interests in treating metastatic and inflammatory diseases (Andexer et al., 2011). Therefore, connecting the shikimate pathway and other pathways with hydroxybenzoate acid as a node may become a powerful strategy for producing valuable bioproducts, including new to nature products.

In conclusion, chromosome-integrated synthetic pathway for GA from 3-HBA were constructed in *Pseudomonas* for the first time based on the enhanced shikimate pathway in P3 strain. The biosynthetic route of GA was constructed by connecting the endogenous degradation pathway and 3-HBA synthetic pathway. This study provides new insights into the possibility of using *Pseudomonas* to synthesize valuable compounds from renewable feedstocks, with a more environmentally responsible, eco-friendly strategy.

## Data Availability Statement

The datasets generated for this study can be found in online repositories. The names of the repository/repositories and accession number(s) can be found in the article/Supplementary Material.

## Author Contributions

SW and XZ conceived and designed the experiments. SW performed experiments, analyzed the experimental data, and drafted the manuscript. CF, KL, and JC assisted in experimental work and manuscript writing. HH and WW contributed reagents & materials. XZ revised the manuscript. All authors contributed to the final paper.

## Conflict of Interest

The authors declare that the research was conducted in the absence of any commercial or financial relationships that could be construed as a potential conflict of interest.
