# Supplementary material for: Engineering a Synthetic Pathway for Gentisate in Pseudomonas Chlororaphis P3
Source: Front Bioeng Biotechnol. 2021 Jan 22;8:622226. doi: 10.3389/fbioe.2020.622226 (PMC7862547; doi:10.3389/fbioe.2020.622226)
Supplement: Supplementary file 1 [file Table_1.DOCX]

**Supporting Information**

Figure S1 UPLC-MS/MS peak spectrum of 3-HBA standard (A) and P3-Hb1 sample (B).

**
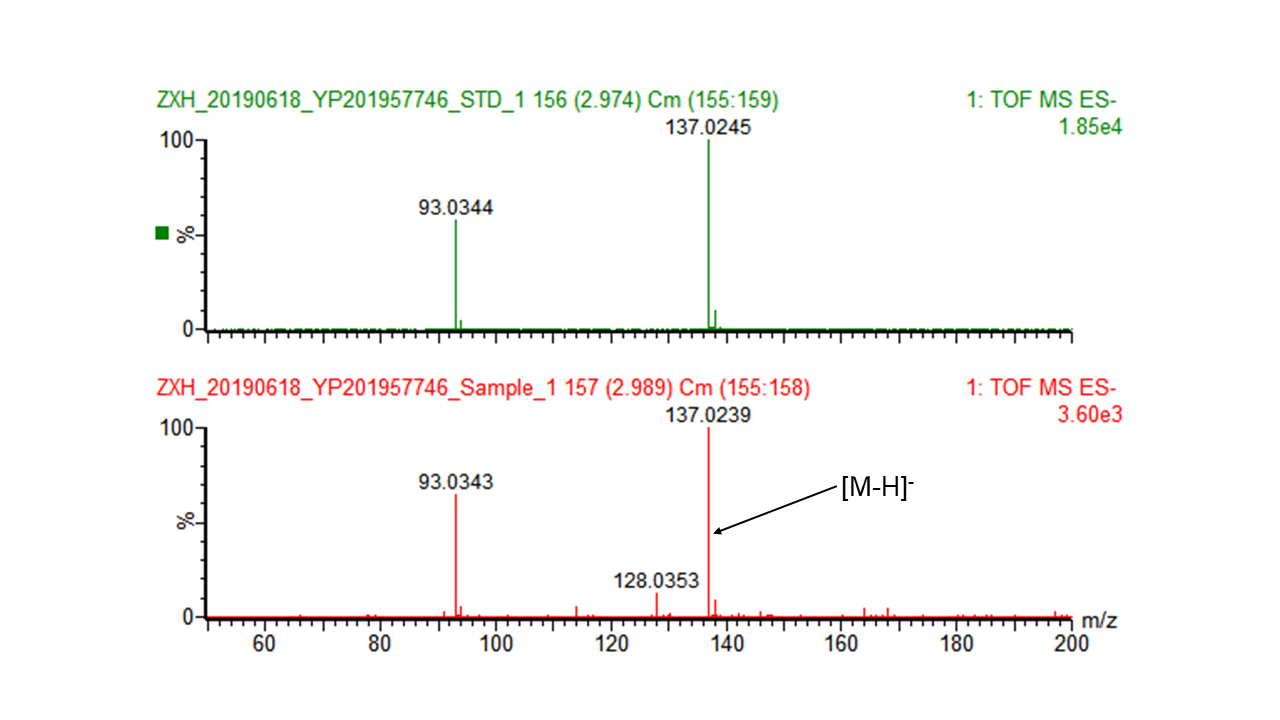
**

**A**

**B**

Figure S2 UPLC-MS/MS peak spectrum of gentisate standard (A) and Sal sample (B).

**
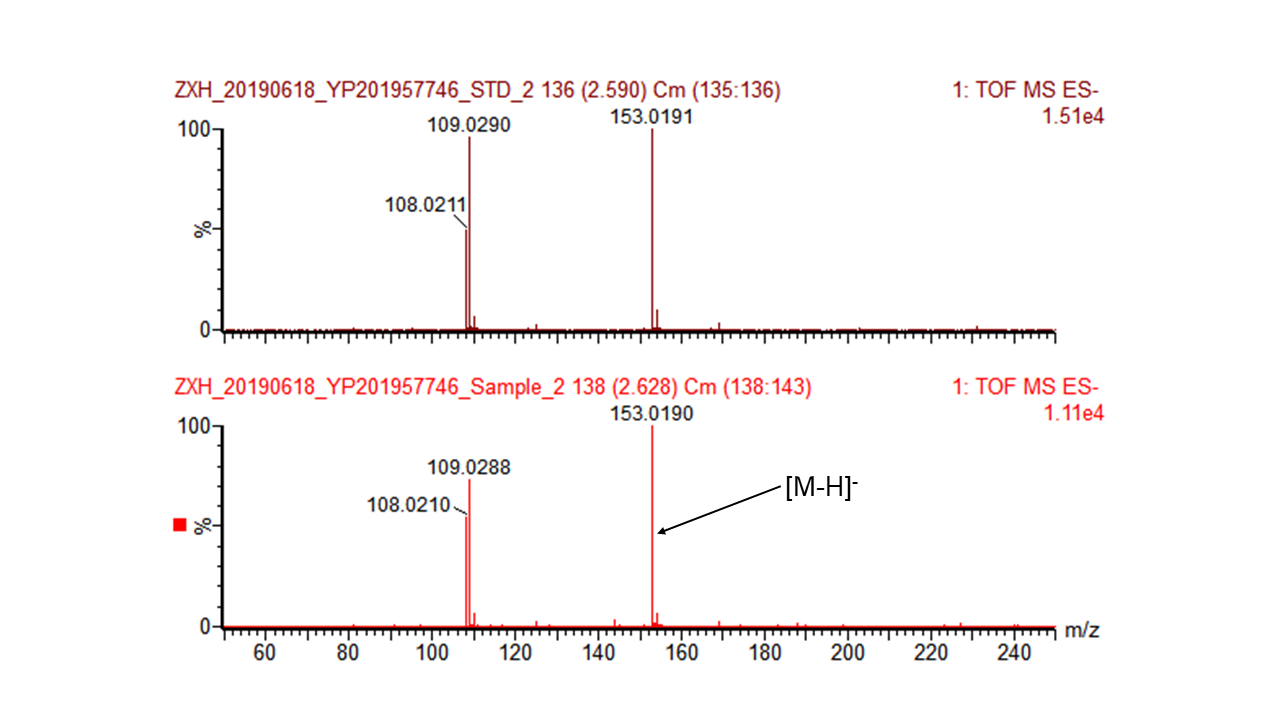
**

**A**

**B**

Figure S3. The concentration of gentisate in each time points. Gentisate with final concentration of 1g/L was added to KB medium and incubated at 28 ℃ for 48 h.


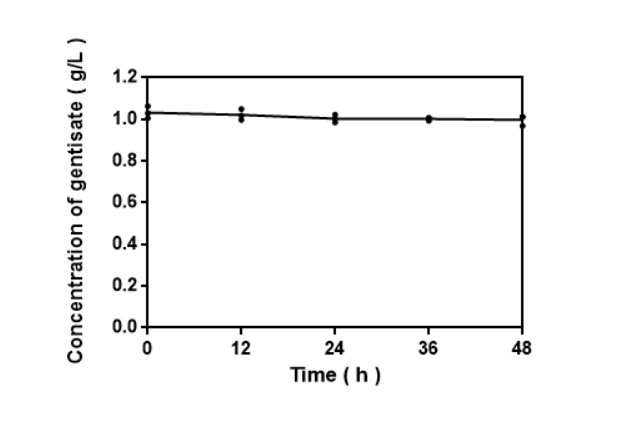


| **Strain** | **Characteristics** | **Source** |
| --- | --- | --- |
| E. coli DH5α λpir | λpir lysogeny of DH5α | Lab stock |
| S17-1 (λ pir) | E. coli res^-^ pro mod^+^ integrated copy of RP4, mob^+^, used for incorporating constructs into *P. chlororaphis* | Lab stock |
| BL21(DE3) | F- ompT hsdSB (rB-, mB-) gal dcm (DE3) | Lab stock |
| *P. chlororaphis P3* | A mutant from *P*. *chlororaphis* HT66 with a high PCN production | Lab stock |
| P3-Hb0 | *P. chlororaphis* P3 with *pykA*, *phzE* deleted, *hyg5* inserted to *phzA*＆*phzB* locus | This study |
| P3-Hb1 | *P. chlororaphis* P3 with *pykA*, *phzE*, *sal* deleted, *hyg5* inserted to *phzA*＆*phzB* locus | This study |
| P3-Hb1m | *P. chlororaphis* P3 with *pykA*, *phzE*, *sal* deleted, *hyg5M* inserted to *phzA*＆*phzB* locus | This study |
| P3-Hbc | *P. chlororaphis* P3 with *pykA*, *phzE*, *sal* deleted, *cuv10* inserted to *phzA*＆*phzB* locus | This study |
| P3-Hhb1 | P3-Hb1, *P_phz_-hyg5 i*nserted to *pykA* locus | This study |
| P3-Hhb2 | P3-Hb1, *P_lac_-hyg5 i*nserted to *pykA* locus | This study |
| P3-Hhb3 | P3-Hb1, *P_lac uv5_-hyg5 i*nserted to *pykA* locus | This study |
| P3-Hhb4 | P3-Hb1, *P_T7_-hyg5 i*nserted to *pykA* locus, T7 RNA polymerase inserted to *phzE* locus | This study |
| P3-Hhb5 | P3-Hb1, *P_tac_-hyg5 i*nserted to *pykA* locus | This study |
| P3-Hhb1△*glpR* | P3-Hhb1, with *glpR* deleted | This study |
| P3-Hhb1△*hexR* | P3-Hhb1, with *hexR* deleted | This study |
| P3-GA1 | *P. chlororaphis* P3 with *pykA*, *phzE*, *mhbD*1 deleted, *hyg5* inserted to *phzA*＆*phzB* locus | This study |
| P3-GA2 | P3-Ga0, *P_phz_-hyg5* inserted to *pykA* locus | This study |
| P3-GA3 | P3-Ga0, with *mhbD2* deleted | This study |
| P3-GA4 | P3-Hga0, with *hgaD* deleted | This study |
| GA-4HBA | *P. chlororaphis* P3 with *pobA*, *phzE*, *mhbD*1 and *hmgA* deleted, *P_phz_-xanB2* inserted to *pykA* locus, *phgA*-*phgB*-*phgC* inserted to *phzAB* locus | This study |
| BL21-Sal | BL21(DE3) harboring pET-*sal* | This study |
| BL21-PobA | BL21(DE3) harboring pET-*pobA* | This study |
| BL21-PobAM | BL21(DE3) harboring pET-*pobAM* | This study |

Table S1 Strains used or engineered in this study

**Table S2 Plasmids used and constructed in this study**

| **Plasmid** | **Characteristics** | **Source** |
| --- | --- | --- |
| pk18*mobsacB* | Broad-host-range gene replacement vector, Km^r^ | Lab stock |
| pET28a(+) | T7 promoter, Km^r^ | Lab stock |
| pk18-△*pobA* | pk18*mobsacB* containing *pobA* upstream and downstream, Km^r^ | This study |
| pk18-△*pykA* | pk18*mobsacB* containing *pykA* upstream and downstream, Km^r^ | This study |
| pk18-△*phzE* | pk18*mobsacB* containing *phzE* upstream and downstream, Km^r^ | This study |
| pk18-△*sal* | pk18*mobsacB* containing *sal* upstream and downstream, Km^r^ | This study |
| pk18-△*mhbD1* | pk18*mobsacB* containing *mhbD1* upstream and downstream, Km^r^ | This study |
| pk18-△*mhbD2* | pk18*mobsacB* containing *mhbD2* upstream and downstream, Km^r^ | This study |
| pk18-△*glpR* | pk18*mobsacB* containing *glpR* upstream and downstream, Km^r^ | This study |
| pk18-△*hexR* | pk18*mobsacB* containing *hexR* upstream and downstream, Km^r^ | This study |
| pk18-△*hgaD* | pk18*mobsacB* containing *hgaD* upstream and downstream, Km^r^ | This study |
| pk18-*hyg5* | pk18*mobsacB* containing *hyg5*, *phzA* upstream and *phzB* downstream, Km^r^ | This study |
| pk18-*hyg5M* | pk18*mobsacB* containing *hyg5M*, *phzA* upstream and *phzB* downstream, Km^r^ | This study |
| pk18-*Cuv10* | pk18*mobsacB* containing *Cuv10*, *phzA* upstream and *phzB* downstream, Km^r^ | This study |
| pk18-*P_phz_*–*hyg5* | pk18*mobsacB* containing *P_phz_–hyg5, pykA* upstream and downstream, Km^r^ | This study |
| pk18-*P_Lac_*–*hyg5* | pk18*mobsacB* containing *P_Lac_–hyg5, pykA* upstream and downstream, Km^r^ | This study |
| pk18-*P_Lacuv5_*–*hyg5* | pk18*mobsacB* containing *P_Lacuv5_–hyg5, pykA* upstream and downstream, Km^r^ | This study |
| pk18-*P_T7_*–*hyg5* | pk18*mobsacB* containing *P_T7_–hyg5, pykA* upstream and downstream, Km^r^ | This study |
| pk18-*P_Tac_*–*hyg5* | pk18*mobsacB* containing *P_Tac_–hyg5, pykA* upstream and downstream, Km^r^ | This study |
| pk18-*T7P* | pk18*mobsacB* containing T7 RNA polymerase, *phzE* upstream and downstream, Km^r^ | This study |
| pk18-*phgABC* | pk18*mobsacB* containing *phgABC*, *phzAB* upstream and downstream, Km^r^ |  |
| pET-*sal* | pET28a(+) containing *sal* | This study |
| pET-*pobA* | pET28a(+) containing *pobA* | This study |
| pET-*pobAM* | pET28a(+) containing *pobAM* | This study |

Table S3 Nucleotides sequences in this study

| Nucleotides | Sequence (5’ → 3’) |
| --- | --- |
| pykA-1F | CATGATTACGAATTC AAGGGTAATGCCCCAGTTGCTGCA |
| pykA-1R | GCAAAGACTCCTGAGTTCAAGCGCA |
| pykA-2F | GCAAAGACTCCTGAGTTCAAGCGCA |
| pykA-2R | GACTCTAGAGGATCC GCCTGCTCGGGCAGGCCAAGG |
| glpR-1F | CATGATTACGAATTC GGCGTGCGTGTGGACCACATCATC |
| glpR-1R | CAGATTCATGGGGCTTCCTTTGGGC |
| glpR-2F | AGCCCCATGAATCTG.CCCCAGCCTCCCCCGTGTAGCC |
| glpR-2R | GACTCTAGAGGATCC GATCAGCTTGCTGCTGGCCGAGGAG |
| hexR-1F | CATGATTACGAATTC TAATCCTCGGCCTTGAGGAAGTCCA |
| hexR-1R | TAGGGTTTTCCTGGTTCGGCAATGC |
| hexR-2F | AACCAGGAAAACCCTAGGCTCGCCTGACCGCTCCTACA |
| hexR-2R | GACTCTAGAGGATCC CACGCCTATTGGCGTATTGGTGACG |
| hyg5-F | ACTCAGGAGTCTTTGC TTTGAGCACCACTAAAGTTGAAAACAGGC |
| hyg5-R | TCACATCACCACGCCCTCGATTTC |
| mhbD2-1F | CATGATTACGAATTC TCCAGGCGTACCGCGTAGTGGG |
| mhbD2-1R | GGCGTGTCGGCCGGGCTATAGC |
| mhbD2-2F | CCCGGCCGACACGCCCAGTTCTCCTGGCCGTGAGTAATAAGTTG |
| mhbD2-2R | GACTCTAGAGGATCC GTCAGGCCCCTCGAGCCGGATT |
| hgaD-1F | CATGATTACGAATTC AGGTCTCGTTCAACTCATCCCGC |
| hgaD-1R | GGAGCCTCCTGGAAGTGGAGTTG |
| hgaD-2F | CTTCCAGGAGGCTCCCCCATGAATCAGCCAACCATCAC |
| hgaD-2R | GACTCTAGAGGATCC TAACCGATCGGCACGTACTTGTAGTT |
| sal-1F | GGCGATCATCGACTACCTGGAT |
| sal-1R | AATGTCACCCATCGGATCGAGT |
| sal-2F | CATGATTACGAATTC GATGCCTTGCATCCCGAGGTAC |
| sal-2R | GACTCTAGAGGATCC GAATGTCACCCATCGGATCGAGTCT |
| TACF | CTCAGGAGTCTTTGCTCATGCCATACCGCGAAAGGTT |
| TACR | AATTGTTATCCGCTCACAATTCCACAC |
| phzhF | ACTCAGGAGTCTTTGC TTTGAGCACCACTAAAGTTGAAAACAGGC |
| phzhR | TCACATCACCACGCCCTCGATTTC |
| T7RF | ATCAAGGAGAAGATCATGAACACGATTAACATCGCTAAGAACGAC |
| T7RR | TTACGCGAACGCGAAGTCCGACTCT |
| rET-1F | CATGATTACGAATTCAGCACCCGATCCTTGCCGTCTT |
| rET-1R | GATCTTCTCCTTGATTGCTTTGTAGGGC |
| rET-2F | TTCGCGTTCGCGTAAGCGTCATGAGCGGCCAGCCC |
| rET-2R | GACTCTAGAGGATCCGCGCGGTGCAGGTAGTCGACGTT |
| psalF | CGCGGCAGCCATATGATGAACCACGAAAAACAACAACAAAAAG |
| psalR | TGCGGCCGCAAGCTTTTACCCCCTCCCCGCCAGAC |
| ppobAF | CGCGGCAGCCATATGATGAAAACCCTGAAA |
| ppobAR | TGCGGCCGCAAGCTTCTACTCGATAGCCTCGTAAG |
| ppobAMF | CGCGGCAGCCATATGATGAAGACTCAAGTCG |
| ppobAMR | TGCGGCCGCAAGCTTCTACTCGATTTCCTCGTAGG |
| mhbD1-1F | CATGATTACGAATTCTCGGCGCCATGCCCGGCGAT |
| mhbD1-1F | GGGACACCTTTGCGAGTTGT |
| mhbD1-1F | TCGCAAAGGTGTCCCACCTTCCGCCCAGACTTTGG |
| mhbD1-1F | GACTCTAGAGGATCCCGTGTTCCAGGCCAGCCAGC |
| phzE-1F | CATGATTACGAATTCCGCTACTTCCTGCGGCCCTT |
| phzE-1R | AGCACCACCTCGTCGGTGGT |
| phzE-2F | ACCACCGACGAGGTGGTGCTCCATGCACCATTACGTCAT |
| phzE-2R | GACTCTAGAGGATCCAACGCGGCGATGCTTGGCA |
| *hyg5* | ATGCTGAACCCGAGCTCGCTCGTGCTGAACGGTTTAACCAGCTACTTCGAAAACGGCCGCGCCCGCGTGGTGCCGCCGGTCGGTCGCAACATTTTAGGCGTCGTGAACTACGCCAGCGTGTGCGAGTACCCGACTTTAGACCACGGCTACCCGGAACTCGAAATCAACATGGTGGCCCCGACGGCCGAACCCTTCGCCGAAGTGTGGGTCACCGACGCCGAAAGCGAGCACGGCGAACGCGACGGTATCACGTACGCCCACGACGGCGAATACTTCTTCTGCGCCGGCCGCGTGCCGCCGACCGGCCGCTATACCGAAGCCACCCGCGCCGCCTACGTGACCATGTTTGAGTTATTAGAGGAGTTCGGCTACAGCAGCGTTTTTCGCATGTGGAACTTCATCGGCGACATCAACCGCGACAACGCCGAAGGTATGGAAGTGTACCGCGATTTCTGCCGCGGCCGCGCCGAAGCCTTCGAACAATGCCGCCTCGAGTTCGATCAGTTCCCGGCCGCCACGGGCATCGGCAGCCGCGGCGGCGGCATCGCCTTCTATTTACTGGCTTGTCGCAGCGGCGGCCACGTGCATATCGAAAACCCGCGCCAAGTTCCCGCGTACCATTACCCGAAGCGGTACGGCCCGCGCGCCCCGCGCTTCGCCCGCGCCACCTATTTACCGAGCCGCGCCGCCGACGGCGTGGGTGGTCAAGTTTTCGTGAGCGGCACGGCGTCGGTGCTGGGCCACGAAACGGCGCACGAAGGCGATTTAGTCAAACAGTGCCGTTTAGCGCTGGAAAACATCGAGCTGGTGATTAGCGGCGGCAATTTAGCCGCCCACGGTATTTCGGCGGGTCACGGTTTAACGGCTTTACGCAACATCAAGGTCTACGTGCGCCGCAGCGAGGATGTGCCCGCCGTGCGCGAAATCTGCCGCGAAGCCTTCAGCCCGGATGCCGACATCGTGTACCTCACCGTGGACGTGTGCCGCAGCGATTTACTGGTGGAAATCGAGGGCGTGGTGATGTGA |
| *sal* | ATGAACCACGAAAAACAACAACAAAAAGTCATCATCGTCGGCGGCGGCATCGGTGGCCTGGCGGCGGCCCTGGCCCTGACCCGCCAGGGCATCCGTGTACAACTGTTGGAACAGGCCGAACAGATTGGCGAGATCGGCGCCGGCATCCAGCTCGGGCCCAATGCCTACGCGGCCCTGGATGCCCTGGGCGCCGGCGAGGCGGCGCGACGCCGTTCAGTGTTCACCGACCACATCATCATGATGGATGCGGTGGACGCTGGCGAAGTGGTGCGCATCGATGTCGGCGCGGCGTTCCAGCAGCGTTTCGGCAACCCCTATGGGGTGATCCACCGCGCCGATATTCACTTGTCGATCCTCGAGGCGGTGGAGCAGGACCCGCTGATCAGCTTCCAGACCTCGACCCGGATTACCAGCATGGACCTGCAAGGGCCCGGAGTGACCCTGATCGACCAGCATGGCCAGCGCTACCAGGCGGACGCGGTGCTGGGCTGCGACGGCGTCAAGTCGGTGGTACGCGAGCAGCTGCTGGGCGATACGCCACGGGTGACCGGGCATGTGGTCTATCGGGCGGTGGTCGACGAGCAGGAGATGCCCGAGGAACTGCGGGTCAACGCCCCGGTGCTGTGGGCCGGTCCCCGTTGCCACCTGGTGCACTACCCATTGCGTGGCGGCCAGCAGTACAACCTGGTGGTGACCTTCCATAGCCGCGAGCAGGAAGAGTGGGGCGTGCGCGACGGCAGCAAGGCCGAGGTGCTGTCGTACTTCCAGGGCATCCACCCGAGCCCGGCCCGGCTGCTGGAGACCCCCAGTTCCTGGCGCCGCTGGGCCACCGCCGACCGCGAGCCGGTAGAACGCTGGGGCCAGGGCAACGCGACCCTGCTCGGCGATGCCGCCCACCCCATGAGCCAGTACCTGGCCCAGGGCGCCTGCATGGCGCTGGAAGACGCGGTGACCCTGGGCGAAGCGGTCAGGGCCTGCGGCCACGACCTGCAGGCGGCGTTCCGCCTCTACGAATCGGTGCGCATCCCCCGCACCGCCCGCGTAGTCTGGTCCACCCGGGAAATGGGCCGCCTCTACCATGCCCAGGGCGTCGAACGCAGCGTGCGCAACAGCCTGTGGGAGGGGCGCAGCCAGGCACAGTTTTATGATGCGGTGCAGTGGTTGTATGGGTGGAATGTCGATAATTGTCTGGCGGGGAGGGGGTAA |
| *pobA* | ATGAAAACCCTGAAAACTCAAGTCGCCATCATTGGCGCCGGCCCCTCCGGACTACTGCTCGGCCAACTGCTGCACAAGGCCGGCATCGATACCCTGATCCTTGAACGGCAAACCCCGGATTACGTACTCGGGCGCATCCGCGCCGGGGTGCTGGAACAAGGCATGGTCGACCTGCTGCGCCAGGCCGGAGTCAGCCAGCGCATGGACGCCGAAGGCCTGGTGCACGGCGGCTTCGAACTGGCCCTCGACGGCCGCCGGGTGGCCATCGACCTGCATGCCCTCACCGGCGGCAAAAGCGTGACGATCTACGGCCAGACCGAAGTCACCCGCGACCTGATGCAAGCCCGCCAGGCCGTCGGCGCCCGGACCCTCTACCAGGCCGACAACGTGCGCCCCCACGACATGCAGAGCGACCAGCCCTACCTGACCTTCGATTACCAGGGCGAGCCCCACCGCCTGGACTGCGACTACATCGCCGGCTGCGACGGTTTCCACGGGGTCGCCCGGCAGTCGATCCCGGCCGACAAGCTCAAGGTTTTCGAGCGCGTCTATCCGTTCGGCTGGCTCGGCATTCTCGCCGATACCCCGCCGGTCCACGACGAACTGGTCTACGCCCGCCACGAACGCGGCTTTGCCCTGTGCAGCATGCGGTCGACCACCCGCACCCGCTACTACCTGCAGGTACCGGCGGAGGAAAATGTCGCCGACTGGTCCGATCAACGCTTCTGGGACGAGCTCAAGAAGCGCCTGCCCGAAGAGCTGGCCGAGCGCCTGGTGACCGGGCCGTCGATCGAGAAAAGCATTGCGCCGCTGCGCAGCTTCGTGGTCGAGCCGATGCAATACGGGCGGATGTTCCTGGTCGGCGACGCCGCGCACATAGTGCCCCCCACCGGAGCCAAGGGCCTGAACCTGGCCGCCAGCGATGTCAGCACACTGTTCAACATTCTGCTCAAGGTCTACCGCGAGCAGCGCACCGACCTGCTGCAGAAGTACTCGGAAATATGCCTGCGGCGGATCTGGAAGGCCGAGCGTTTCTCCTGGTGGATGACCGCGATGCTGCACCGTTTCCCCGACTCCGACGACTTCAGCCAGCGCATCGCCGAGTCGGAGCTGGCGTACTTCGTCGACTCCGAGGCCGGGCGCAAAACCATCGCGGAAAATTACGTCGGCCTTCCTTACGAGGCTATCGAGTAG |
| *pobAM* | ATGAAGACTCAAGTCGCCATCATCGGCGCCGGTCCGTCCGGCCTCCTGCTCGGCCAGTTGCTGCACAAGGCCGGCATCGACAACGTGATCCTCGAACGCCAGACCCCGGACTACGTGCTCGGCCGCATCCGCGCCGGCGTGCTGGAACAGGGTATGGTCGACCTGCTGCGCGAGGCCGGCGTCGACCGGCGCATGGCGCGCGACGGGCTGGTCCACGAAGGCGTGGAGATCGCCTTCGCCGGGCAGCGCCGGCGCATCGACCTGAAGCGCCTGAGCGGCGGCAAGACGGTGACGGTCTACGGCCAGACCGAGGTCACCCGCGACCTCATGGAGGCCCGCGAAGCCTGCGGCGCCACTACCGTCTACCAGGCCGCCGAGGTGCGCCTGCACGACCTGCAAGGTGAGCGCCCCTACGTGACCTTCGAACGCGACGGCGAACGGCTGCGCCTGGATTGCGACTACATCGCCGGCTGCGATGGCTTCCACGGCATCTCGCGGCAATCGATCCCGGCGGAGCGGCTGAAGGTCTTCGAGCGGGTCTATCCGTTCGGCTGGCTCGGCCTGCTCGCCGACACCCCGCCGGTGAGCCACGAACTGATCTACGCCAACCATCCGCGCGGCTTCGCCCTGTGCAGCCAGCGTTCGGCCACCCGCAGCCGCTACTACGTGCAGGTGCCATTGTCGGAGAAGGTCGAGGACTGGTCCGACGAGCGCTTCTGGACGGAACTGAAGGCGCGACTCCCGTCCGAGGTGGCGGAGAAACTGGTGACCGGACCTTCGCTGGAGAAGAGCATCGCGCCGCTGCGCAGCTTCGTGGTCGAGCCGATGCAGCATGGCCGGCTGTTCCTCGCCGGCGACGCCGCGCACATCGTGCCGCCCGCCGGCGCCAAGGGACTGAACCTGGCCGCCAGCGACGTCAGCACGCTCTACCGGCTGCTGCTGAAGGCCTACCGCGAAGGGCGCGGCGAACTGCTGGAACGCTACTCGGCAATCTGCCTGCGGCGGATCTGGAAGGCCGAACGCTTCTCCTGGTGGATGACTTCGGTGCTGCATCGCTTCCCCGACACCGACGCGTTCAGCCAGCGCATCCAGCAGACCGAACTGGAGTATTACCTGGGCTCCGAGGCGGGCCTGGCGACCATCGCCGAGAACTTTGTCGGCCTGCCCTACGAGGAAATCGAGTAG |
